# Efficacy of human resource development program for young industry personnel who will be involved in future medical device development

**DOI:** 10.1007/s00464-023-10474-3

**Published:** 2023-10-27

**Authors:** Yumi Tsugita, Yuki Ushimaru, Takamitsu Kato, Motoki Sasaki, Taishi Hata, Makoto Hosaka, Hidetoshi Eguchi, Yuichiro Doki, Kiyokazu Nakajima

**Affiliations:** 1 Department of Next Generation Endoscopic Intervention (Project ENGINE), Osaka University Graduate School of Medicine, Osaka, Japan; 2 Department of Gastroenterological Surgery, Osaka University Graduate School of Medicine, Osaka, Japan; 3 Department of Next Generation Endoscopic Intervention (Project ENGINE), Center of Medical Innovation and Translational Research, Osaka University Graduate School of Medicine, Suite 0912, 2-2, Yamadaoka, Suita, Osaka, 565-0871 Japan

**Keywords:** Education, Research and development, Human resource

## Abstract

**Background:**

Training next-generation personnel from small/medium enterprises (SMEs) is an urgent issue in promoting medical device research and development (R&D). Since 2014 we have engaged in governmentally funded human resource development program for medical/non-medical SMEs, and have assessed its effectiveness by analyzing self-evaluation of achievement level (SEAL) data obtained before and after the training course.

**Methods:**

Human resource development experts interviewed 34 key opinion leaders with deep knowledge of medical device R&D from industry, government, and academia. The skills required for R&D personnel were written down, and a set of skills was created by making a greatest common measure in the list of common elements among them. Using that skill sets, skill evaluations were conducted on trainees at “Osaka University Training Course,” twice before participation and after completion of the entire program using SEAL assessment.

**Results:**

There were 97 men and 25 women, with one-third in the’30 s. Among them, 61 participants (50%) were from R&D divisions, and 32 (26%) were from business/sales divisions. 94 (77%) were from medical SMEs, and 28 (23%) were from non-medical SMEs (new entry). After completing the training course, significant growth was observed in every item of both Soft and Hard skill sets. Especially in new entry SME members, a striking improvement was observed in practical medical knowledge to enhance communication with medical doctors (*p* < 0.0001).

**Conclusion:**

Our training course, though 7-day-short in total, showed that both Soft and Hard skills could be improved in young medical/non-medical SME members. Further assessment is needed to establish the necessary skill sets for our future partners from industries, to foster the creation of innovative medical devices through med-tech collaboration.

Physicians identify almost all unmet needs in the clinical field, yet it’s the business professionals who actualize these needs into tangible research and development (R&D) endeavors in the medical devices [1–5]. The challenge? Most physicians aren’t entrenched in the design, manufacturing, or business aspects. Therefore, the industry’s answer has been to train “development personnel” adeptly for medical device R&D [6].

While global institutions like Stanford University’s Biodesign Program or Minnesota’s Medical Device Innovation Experience for Undergraduates (MDIEU) focus on intensive, prolonged courses [7–9], Japan has innovatively veered in different direction.

The Japan Agency for Medical Research and Development (AMED) launched “Human Resource Development Project” in 2014. We Osaka University, since its inception, has device our original course for personnel from small/medium enterprises (SMEs), conducting R&D skill mastery into mere weeks. Uniquely, no other course globally offers such a compact curriculum. Now, with our bespoke “skill evaluation system,” we’re set to validate this novel approach.

In our original training courses, we aimed to (1) identify essential skills for “development personnel” and establish a “skill evaluation system,” and (2) assess the effectiveness and improvement of our course through this system. Our study evaluates skill enhancement in our unique, concise training program.

## Methods

### Identification of required skill sets and establishment of a skills assessment system

**Fig. 1:**
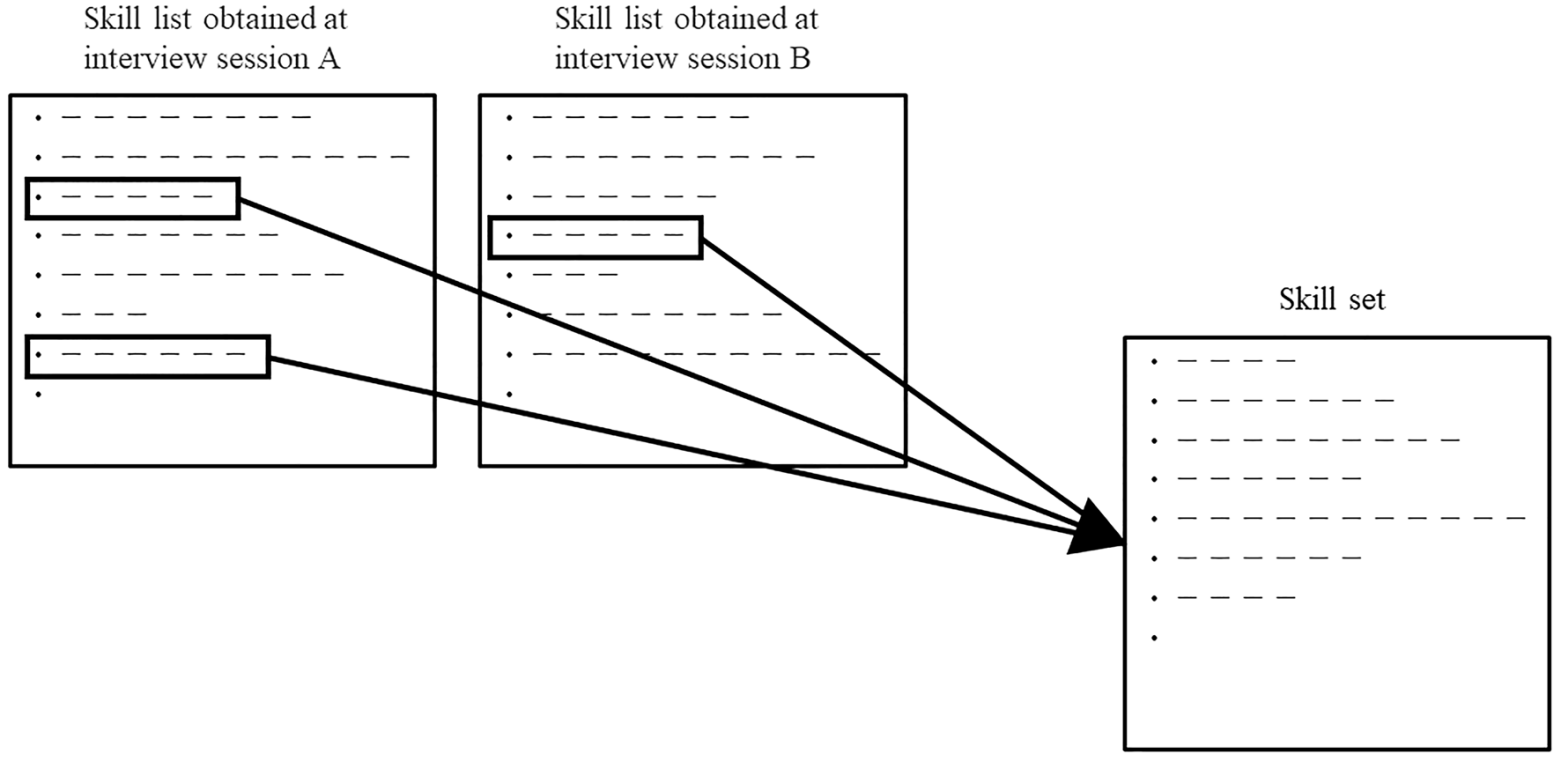
Methodology of skill set creation

In the early stage of the project (first or second year of the program), we first worked on the establishment of a skill evaluation system. Human resource development experts interviewed 34 key opinion leaders (KOLs) with deep knowledge of medical device R&D from industry, government, and academia (Table 1). This is separate from the participants who took part in the Osaka University Training Course. The skills required of medical device R&D personnel [10] were written down, and a set of skills was created by making a greatest common measure in the list of common elements among them (Fig. 1).

**Table 1:** Breakdown of 34 KOLs interviewed to build skills assessment system

Medical device manufacturer	Manager and above	8 (23.5%)
	Newcomer	3 (8.8%)
Health care provider	Medical doctor	9 (26.5%)
	Clinical engineer	1 (2.9%)
Government	Official, health care administrator	6 (17.6%)
Industry-Academia collaboration	Business consultants	2 (5.8%)
	University faculty	1 (2.9%)
	Lawyer	1 (2.9%)
Academia	Medicine	1 (2.9%)
	Engineering	2 (5.9%)
Total		34 (100%)

### Skill assessment before/after the training course using a skill assessment system

**Fig. 2:**
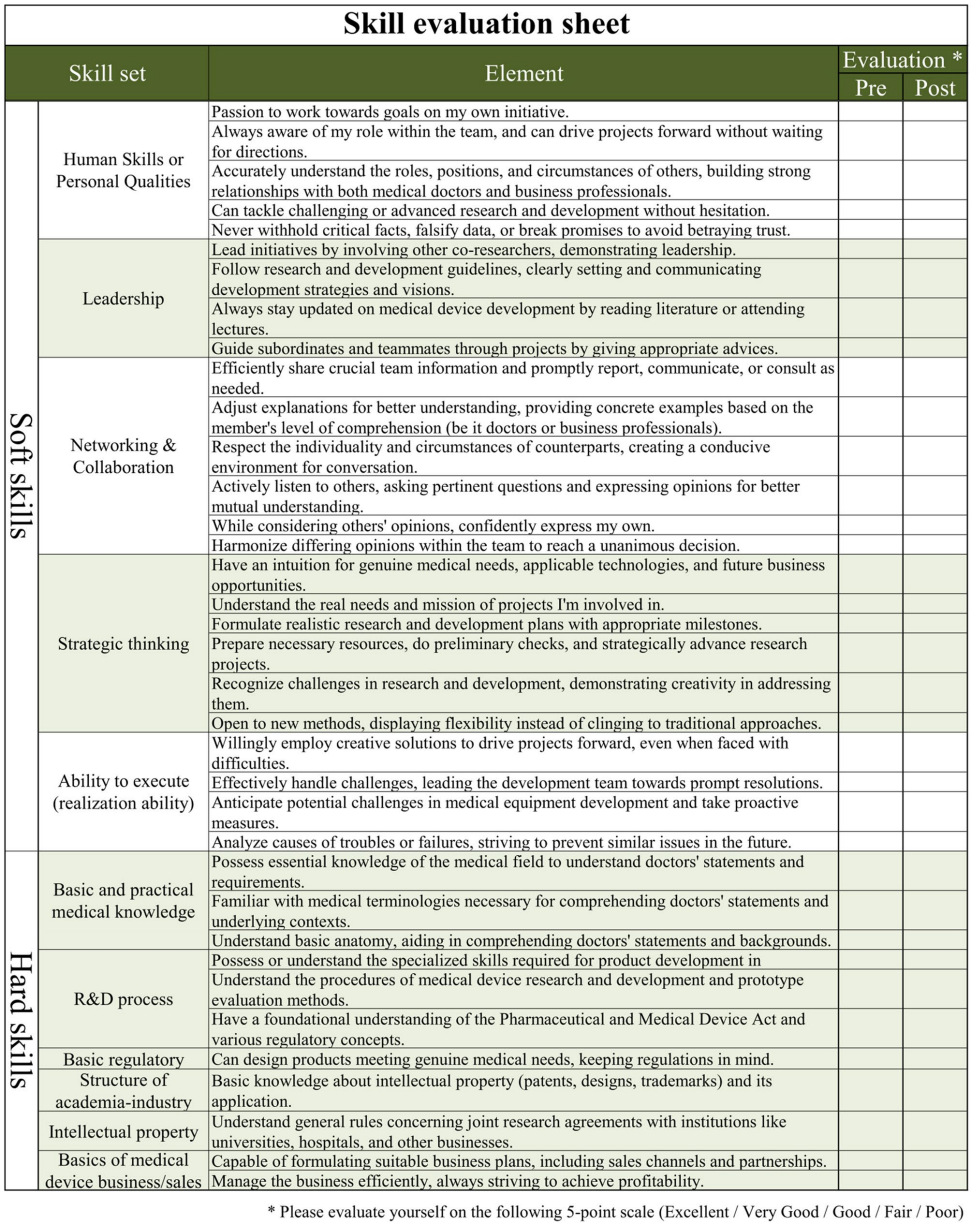
Skill evaluation sheet

To evaluate the efficacy of human resource development, we utilized the skill sets identified in Method 1, skill evaluations were conducted on participants at “Osaka University Training Course,” twice: before participation and after completion of the entire program, using a self-scoring system “self-evaluation of achievement level” (SEAL). To ensure consistency and enable a direct comparison of the participants’ skills before and after the training, the same questionnaire was used for both pre-training and post-training evaluations (Fig. 2).

#### Osaka University Training Course

This is a 7-day training course consisted of 4 programs: 2 days of seminars, 3 days of clinical immersion, 1 day of round table ideation workshop, and 1 day of interactive symposium.

#### Seminars

**Fig. 3:**
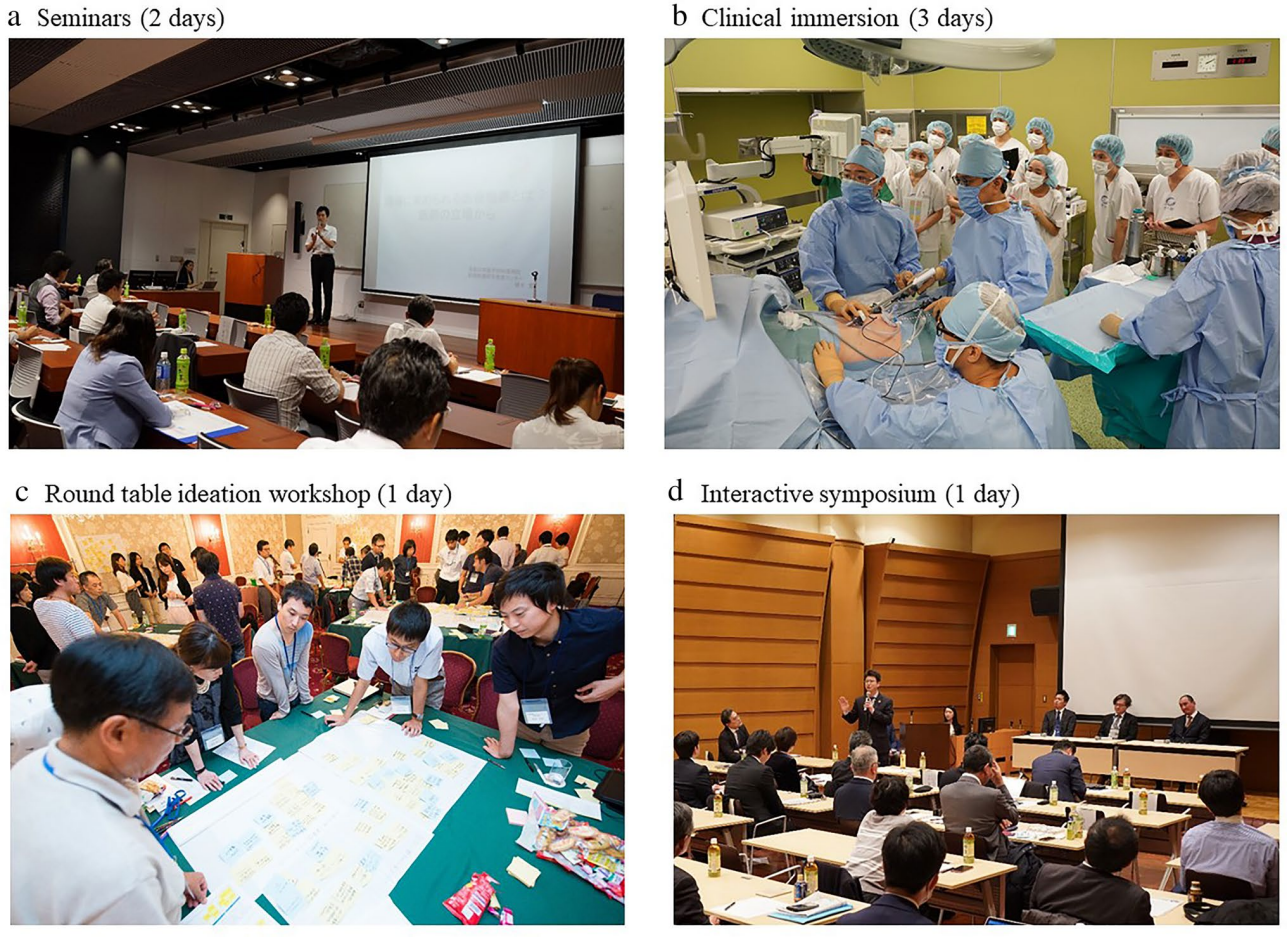
Practical training situations. **a** Seminars (2 days), **b** clinical immersion (3 days), **c** round table ideation workshop (1 day), **d** interactive symposium (1 day)

Faculty members gave lectures on broad topics, e.g., an overview of the medical device business, needs finding, regulatory processes, quality management system (QMS), marketing, and intellectual property (IP) issues. Active surgeons from Osaka University Hospital also talked about basic anatomy, some technical terms, and topic, e.g., the current situation of operating room (OR). We also organized “hands-on” seminars for attendees to touch actual medical devices (Fig. 3a).

#### Clinical immersion

**Fig. 4:**
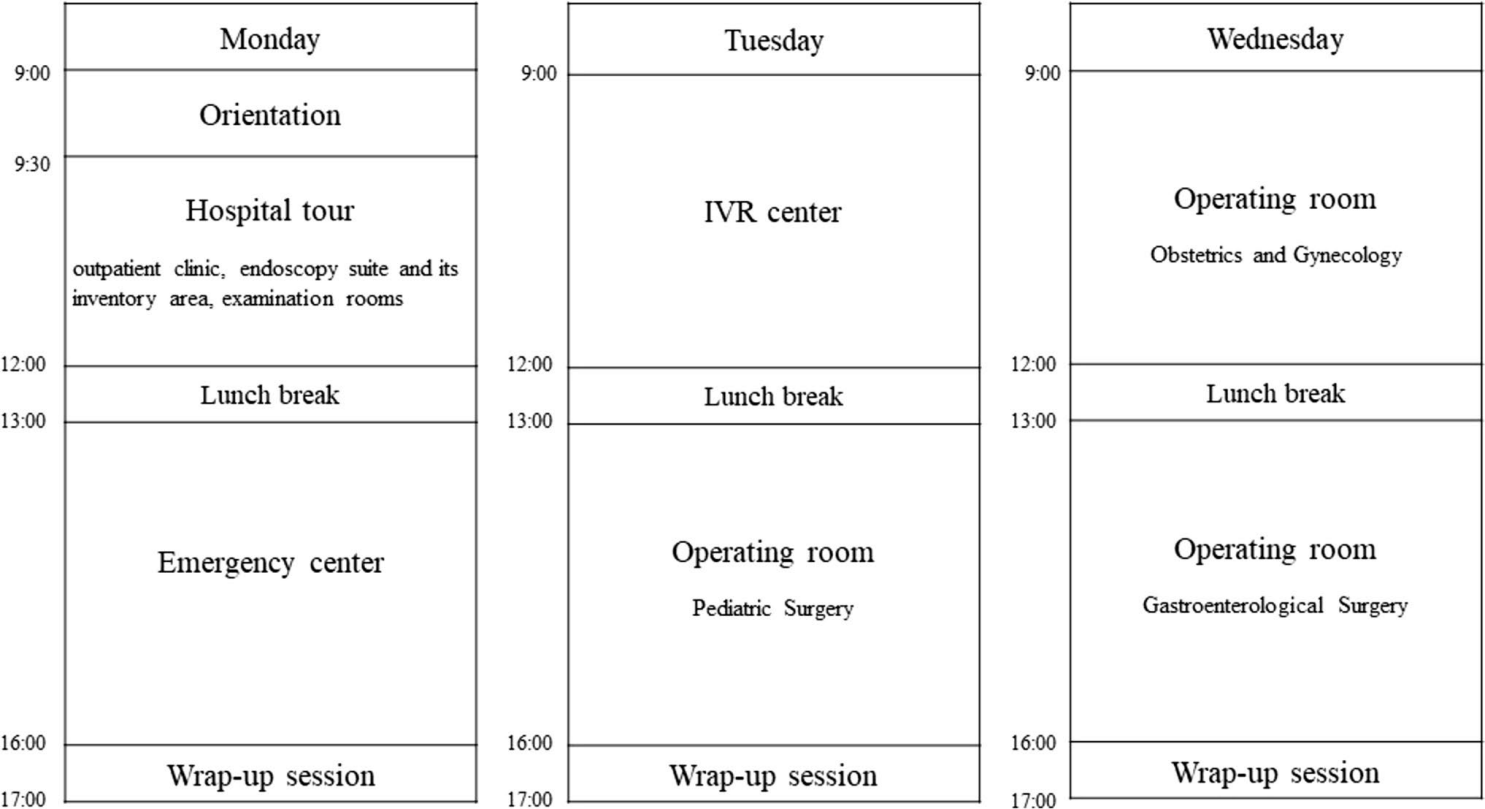
Clinical immersion session schedule

Over a period of three days, segmented into morning and afternoon sessions, we embarked on a comprehensive hospital tour (Fig. 4). The participants were then allowed to go into deeper areas of the hospital, e.g., the backyard of the outpatient clinic, the endoscopy suite and its inventory area, examination rooms, and OR. With the collaboration of the subspecialty teams, we had the opportunity to observe cases in gastroenterological, obstetrics and gynecology, and pediatric surgery. Our exploration further led us to the radiology department, specifically the IVR center, and the emergency medical center. The primary focus of our tour was departments utilizing medical equipment. Care was taken to ensure our presence did not interfere with any ongoing treatments, and we endeavored to expose our participants to a wide array of medical scenarios. At the end of each immersion day, surgeons and attendees had a one-hour wrap-up session to discuss their insights and questions (Fig. 3b).

#### Round table ideation workshop

The trainees were divided into several teams based on their professions, age, gender, etc. This team division was carefully conducted under the supervision of the program director to create intragroup “diversity.” Each team was attended by one physician from Osaka University Hospital, and one professional facilitator. Raw ideas and rough insights were once spread and consolidated using the latest facilitation methodology (Fig. 3c).

#### Interactive symposium

The above achievements were presented by faculty members and participants at half-day symposia. A special session was programmed to discuss “how to facilitate the idea output process.” (Fig. 3d).

### Evaluation of participant’s impressions

**Fig. 5:**
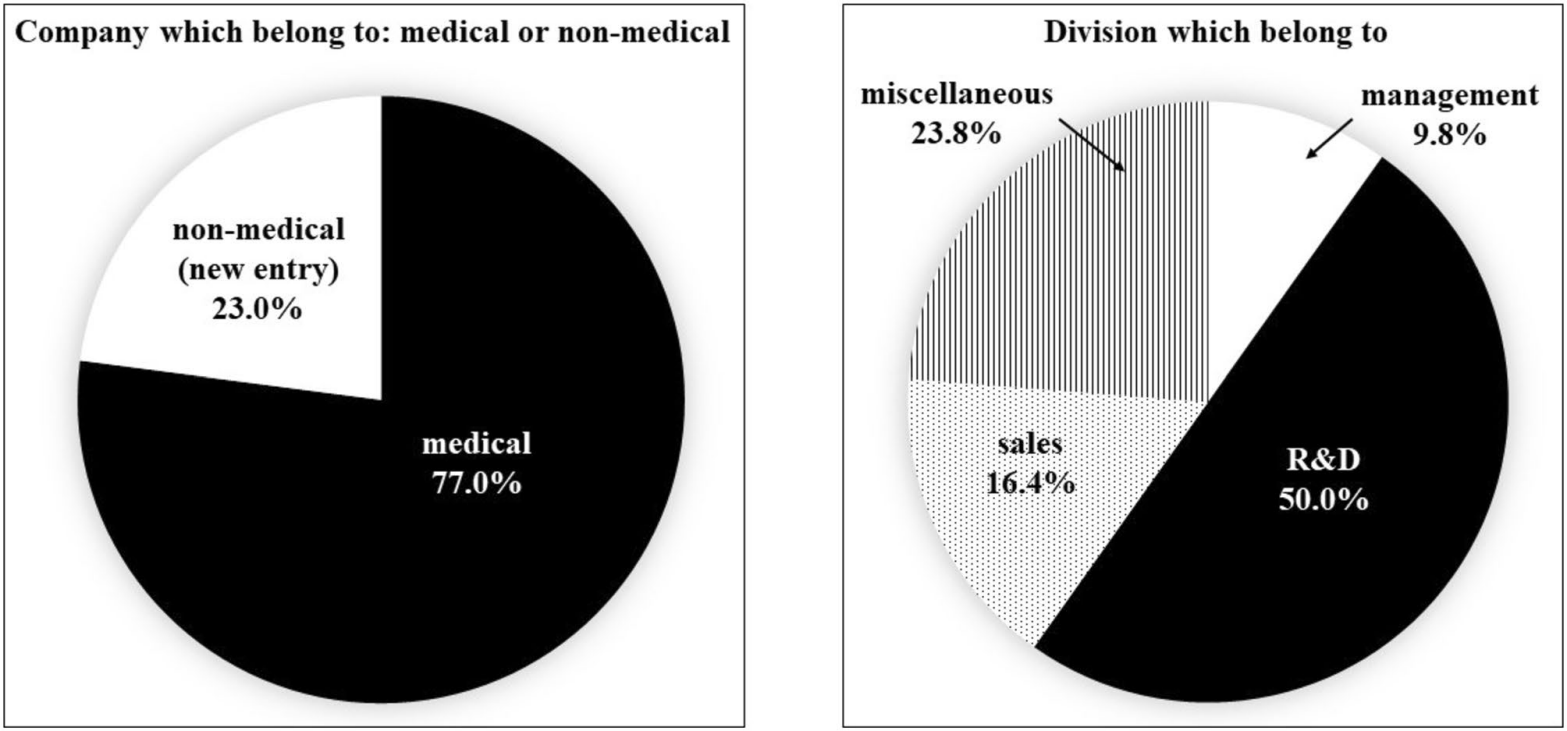
Background of trainees. Participant’s company industry and division

A structured questionnaire was provided to gauge the participant’s impression to each segment of the course: satisfaction, somewhat satisfied, somewhat dissatisfied, complain (Fig. 5).

#### Statistical analysis

The SEAL data were prospectively compiled and retrospectively analyzed. The statistical analyses were performed using the software package (JMP version 16.0.0, SAS, Cary, NC, USA). The results were presented as average ± standard deviation. Student’s *t*-test or paired-samples *t*-test were used to compare continuous variables, respectively. All values were two-tailed, and *p* values of < 0.05 were considered statistically significant.

## Results

For the third through fifth years of the AMED training program, a total of 122 participants from 50 companies took part in the Osaka University Training Course, separate from the 34 key opinion leaders interviewed. The breakdown was 97 males and 25 females, 94 (77.0%) were in the medical field, 28 (23.0%) were non-medical (new entry). 12 (9.8%) were in management, 61 (50.0%) were in development, 20 (16.4%) were in sales divisions, and 29 (23.8%) were in other industries (Fig. 2).

### Identification of required skill sets and establishment of a skills assessment system

As a result of interviews with 34 KOLs and data analysis, a total of 138 skills were listed and 36 of them were determined to be essential skill sets as common denominators. These skills were divided into two subsets: 11 “Hard skills” that could be mastered through classroom lectures, books, video materials, etc., and 25 “Soft skills” that are difficult to master through classroom lectures and books, such as communication skills with doctors and leadership skills within a team, and can be improved mainly through experience (Table 2).

**Table 2:** Skill sets for skill rating system

	Skill categories	# items
Soft skills (25 items)	Human power	5
	Leadership	4
	Networking ability	6
	Strategic thinking ability	6
	Energy (realization ability)	4
Hard skills (11 items)	Basic and practical medical knowledge^a^	3
	R&D process	3
	Basic regulatory science	1
	Structure of academia-industry collaboration	1
	Intellectual property	1
	Basics of medical device business/sales	2

^a^Knowledge involving anatomy and other medical terminology

### Skill assessment before/after the training course using a skill assessment system

Table 3 show how each skill item changed before and after the course. Both the overall score, Soft skills and Hard skills showed significant improvement after completion of the entire program compared to before participating in the course. Each subgroup also showed significant improvement after completion of the entire program compared to before participating in the course (Table 3).

**Table 3:** Assessment results

		Before	After	*p*
Soft skills (25 items)	Human power	15.9 ± 3.0	16.9 ± 3.0	0.013
	Leadership	10.8 ± 3.0	11.9 ± 2.5	0.002
	Networking ability	15.6 ± 4.4	16.9 ± 4.3	0.016
	Strategic thinking ability	14.7 ± 3.8	16.6 ± 3.7	< 0.001
	Energy (realization ability)	10.4 ± 2.5	11.5 ± 2.4	< 0.001
Hard skills (11 items)	Basic and practical medical knowledge	4.9 ± 2.2	6.5 ± 2.3	< 0.001
	R&D process	6.2 ± 2.3	7.2 ± 2.3	< 0.001
	Basic regulatory science	1.7 ± 0.7	2.1 ± 0.8	< 0.001
	Structure of academia-industry collaboration	2.0 ± 0.9	2.4 ± 0.9	0.002
	Intellectual property	2.9 ± 1.0	2.4 ± 1.0	0.003
	Basics of medical device business/sales	4.3 ± 1.8	4.8 ± 1.8	0.030

### Evaluation of participant’s impressions

**Fig. 6:**
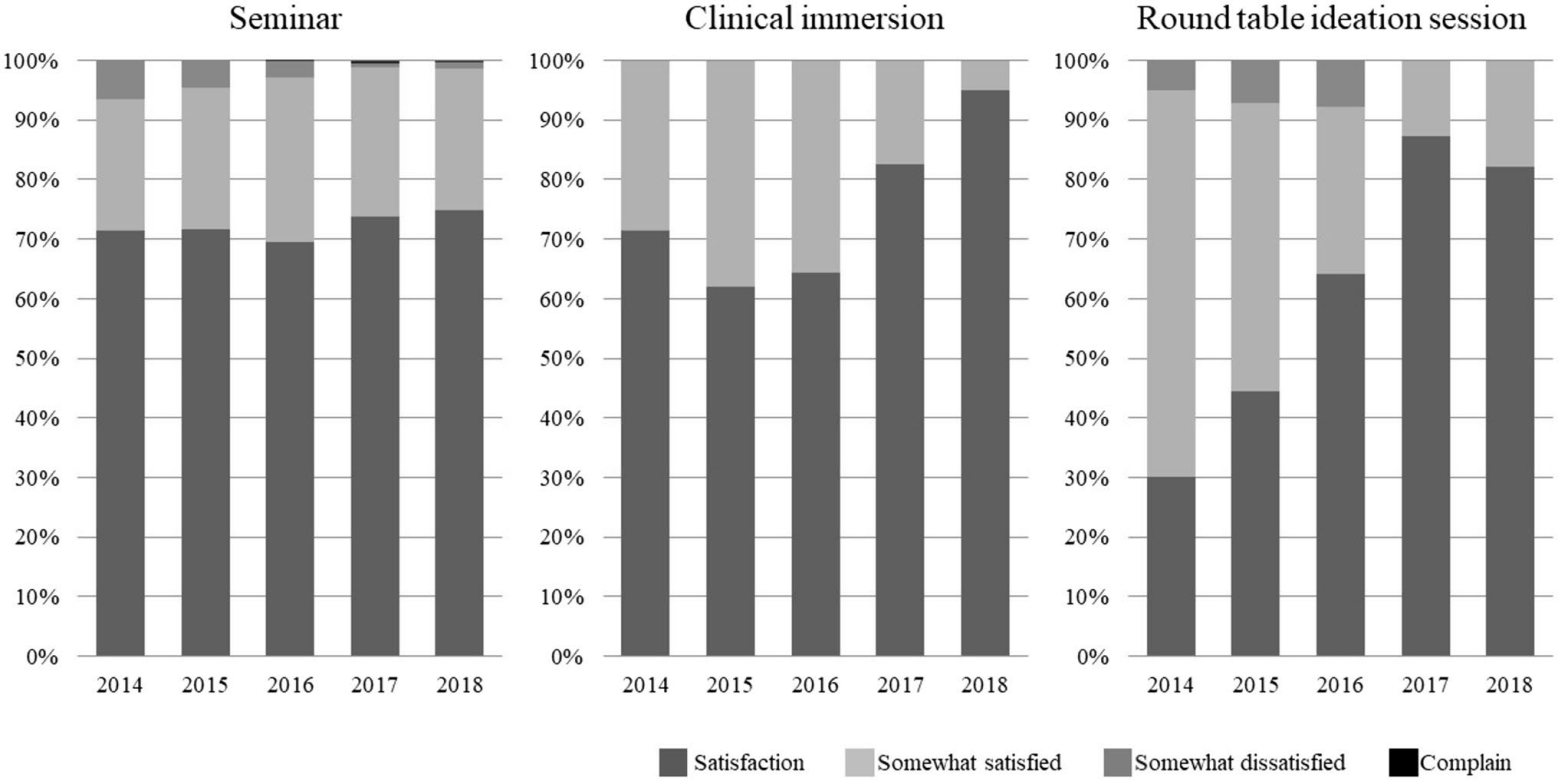
Questionnaire results

Figure 6 presents the aggregated feedback from participants across the seminar, clinical immersion, and needs assessment sessions. The data reveals that about 70% of participants expressed satisfaction with both the seminar and clinical immersion, while an additional 30% indicated they were somewhat satisfied. For the needs assessment session, satisfaction was even higher, with approximately 80% of participants expressing satisfaction and 20% being somewhat satisfied. These results underscore the overall positive reception and effectiveness of the training modules.

## Discussion

The starting point for medical device R&D is the medical field, and for this reason it is believed that physicians occupy an important position as a main player, but in reality, the role played by industry personnel is no less significant. What is especially needed are industry personnel who have a common language with physicians and can discuss issues on an equal footing. In the real world, there is a big gap between doctors and industry people. Industry people tend to take doctors at their word and accept without questioning [11]. They are unable to actively express their opinions and tend to be passive in discussions with doctors, who are medical professionals [12]. As a result, they are often greatly influenced by the opinions of physicians and sometimes mislead. It goes without saying that in order for both sides to be able to have fair discussions on an equal footing as much as possible, it is necessary for physicians to be “more accommodating to the industry side,” but industry people must also master basic medical knowledge (Hard skills) and acquire the Soft skills to communicate with physicians as professionals or specialists.

The gap that hinders the R&D of medical devices is not limited to the gap between physicians and industry people, but actually exists within their companies as well. Even within the companies, there is often a lack of communication between designers/engineers working in the design/development divisions and the business people in charge of commercialization. In other words, there are often cases where the development side lacks understanding of regulations and the commercialization process, and the business side lacks sufficient understanding of the true significance of the developed product. The business people who lead medical device R&D need not only knowledge of regulations, QMS and IPs (Hard skills), but also Soft skills are required, such as coordinating opinions, exercising leadership, and coordinating with physicians across gaps in job types and divisions within the company or development team to which they belong.

Until now, there has been a vague understanding that industry personnel engaged in medical device R&D are required to have these skills, which are classified as Hard skills and Soft skills, but the specifics of the skill sets had not been clarified. We succeeded for the first time in “visualizing” the contents of this “invisible” skill set, and in constructing a “skill evaluation system” based on these skills. Using this system, we have also demonstrated for the first time that both Hard and Soft skills can be improved through an intensive program that combines seminars, group learning, and presentations with clinical practice at its core, even in a very short period of time (one week in total).

Although our training course was extremely short, it was reasonably effective. What was particularly unexpected was that not only Hard skills but also Soft skills improved in a short period of time. The reasons for this can be attributed to the following: (1) about half of the course (3 days) was devoted to clinical immersion, during which participants were closely followed by physicians from several departments, allowing them to focus on observing and thinking about the field from their own perspective; (2) physicians were always present during programs other than training and always engaged in close dialogue with the participants, which allowed them to engage in discussions without hesitation; (3) the course was designed to be a “hands-on” experience for the participants. (4) The latest design thinking process was introduced in the ideation workshop so that participants could experience the diffusion and convergence process of ideas under the supervision of a professional facilitator. This helped them to develop confidence in their ability to carry out the mission of developing medical devices, which seemed to be a high hurdle to overcome. (5) To improve Hard skills, an original glossary of medical terminology was created and video materials (e-learning) were produced for quick learning, so that busy industry people could complete the course without losing motivation.

Industry people engaged in the R&D of medical devices should make every effort to improve these Hard and Soft skills to narrow the gap with physicians as much as possible. Our study suggests that a very short “human resource development course” for this purpose would be reasonably effective. But is it enough? Of course not. Doctors must also be more accessible to the industry side. In some cases, it may be effective for physicians to enter the industry side (become an industry person). In fact, in Europe and the States, human resources are highly mobile, and there are many cases of physicians taking the lead in development at medical device manufacturers and other companies. There are also many doctors who start up their own businesses. In Japan, on the other hand, doctors live only in the medical world, while business people live only in the corporate world, and there is almost no interaction between the two. This rigid situation (low mobility of human resources) may be one of the reasons for the 1 trillion yen excess of imports of medical devices in Japan.

Now, whether or not our human resource development course was truly effective for medical device R&D, should be judged not only by whether or not the participants' skills improved, but also by whether or not a new medical device R&D project was actually started and, by extension, whether or not a real medical device was completed and released to the market (implemented in society) [13]. In reality, it is not easy to judge these true outcomes of this project, since these processes require a long period of in-depth follow-up to confirm the results. We were not able to confirm such a long-term process in this case. It is thought that it may have been possible to identify, albeit indirectly, what skills were acquired through SEAL. However, it is impossible to know whether human resources were truly developed. Skills may have been improved, but it is not clear whether those skills really contributed/lead to medical device R&D. However, several companies that participated in our course have confirmed that they have started medical device R&D in collaboration with universities, launched in-house development projects, and so on. We believe that the course we have established is not only improving the skills of individuals, but is also promoting medical device R&D itself.

There are several limitations in our study. First, we have not been able to evaluate the validity of the skill evaluation system itself. Although KOLs of medical device R&D in Japan participated in the composition of the skill assessment system, we have not been able to evaluate its validity. The second is that the scoring of the skill assessment was self-scoring (SEAL). Although skill assessments should ideally be administered by others, many of the content was difficult or virtually impossible for others to assess. Therefore, self-assessment was employed in this study. In the future, the development of a scoring system by a third party will be required. The third is that our study is retrospective. We examined the assessment of whether the skills were improved backward rather than forward looking. Lastly, our course was free as it was an AMED-subsidized project. We anticipated the possibility that free of charge would lead to a bias against participants, resulting in lax evaluations and motivation. In the future, the issue will be how similar courses are evaluated by participants in self-sustaining projects that charge a certain participation fee. We have clarified our data presentation for transparency and highlighted our study's unique advantages. However, we acknowledge the limitations, especially the reliance on self-assessment. While our study provides evidence of skill enhancement through consistent pre- and post-training evaluations using the same questionnaire, we recognize the potential limitations and biases that might arise from self-assessments. Future iterations of this research could benefit from incorporating diverse assessment tools to provide a more comprehensive understanding of the skills acquired and areas of improvement.

## Conclusion

We have successfully implemented a very short training program for industry personnel who will engage in medical device R&D. Though 1-week short, our program showed reasonable effectiveness in improving their skills. While our findings suggest the effectiveness of our ultra-short training program, we recommend future studies to incorporate detailed pre-and-post training surveys to further validate and understand the nuances of human resource development in the context of medical device R&D. Similar efforts need to be further utilized to promote medical device R&D in the future.
